# Updates on Osteoimmunology: What's New on the Cross-Talk Between Bone and Immune System

**DOI:** 10.3389/fendo.2019.00236

**Published:** 2019-04-18

**Authors:** Marco Ponzetti, Nadia Rucci

**Affiliations:** Department of Biotechnological and Applied Clinical Sciences, University of L'Aquila, L'Aquila, Italy

**Keywords:** osteoimmunology, RANKL, osteoclasts, osteoblasts, immune cells, inflammation, rheumatoid arthritis, osteoporosis

## Abstract

The term osteoimmunology was coined many years ago to describe the research field that deals with the cross-regulation between bone cells and the immune system. As a matter of fact, many factors that are classically considered immune-related, such as InterLeukins (i.e., IL-6, -11, -17, and -23), Tumor Necrosis Factor (TNF)-α, Receptor-Activator of Nuclear factor Kappa B (RANK), and its Ligand (RANKL), Nuclear Factor of Activated T-cell, cytoplasmatic-1 (NFATc1), and others have all been found to be crucial in osteoclast and osteoblast biology. Conversely, bone cells, which we used to think would only regulate each other and take care of remodeling bone, actually regulate immune cells, by creating the so-called “endosteal niche.” Both osteoblasts and osteoclasts participate to this niche, either by favoring engraftment, or mobilization of Hematopoietic Stem Cells (HSCs). In this review, we will describe the main milestones at the base of the osteoimmunology and present the key cellular players of the bone-immune system cross-talk, including HSCs, osteoblasts, osteoclasts, bone marrow macrophages, osteomacs, T- and B-lymphocytes, dendritic cells, and neutrophils. We will also briefly describe some pathological conditions in which the bone-immune system cross-talk plays a crucial role, with the final aim to portray the state of the art in the mechanisms regulating the bone-immune system interplay, and some of the latest molecular players in the field. This is important to encourage investigation in this field, to identify new targets in the treatment of bone and immune diseases.

## Introduction

Evidence collected over the years draw bone researchers to the conclusion that bone accomplishes several unexpected functions besides its classical role in locomotion, protection of vital organs and in the regulation of calcium and phosphate homeostasis. As a matter of fact, it is now well-accepted that bone has a role in the regulation of glucose metabolism, energy expenditure (1–3), male fertility and cognitive functions, through osteoblasts secretion of osteocalcin (4). Therefore, we can assume that bone is a central organ, capable of regulating several other tissues and to be in turn influenced by them.

An intriguing aspect testifying the versatility of bone and its cells, is its deep cross-talk with the immune system. This led to the establishment of a new interdisciplinary field, named osteoimmunology, thanks to the great contribution of studies by Takayanagi and many others. These pointed out the pivotal role of some classical immunoregulatory factors in osteoclast differentiation (5) as well as the cross-talk between autoimmune diseases, such as Rheumatoid Arthritis (RA), and bone destruction (6). Intriguingly, the cross-talk between the immune system and bone is bidirectional, meaning that bone cells also influence immune cells.

In this review, we will describe the main milestones in the history of osteoimmunology, as well as the latest findings enriching this discipline, with the final aim to have a state-of-the-art reference of the mechanisms regulating the bone-immune system interplay. This is important to encourage investigation in this field, in order to identify new targets in the treatment of bone and immune diseases.

## Bone Biology

**Figure 1:**
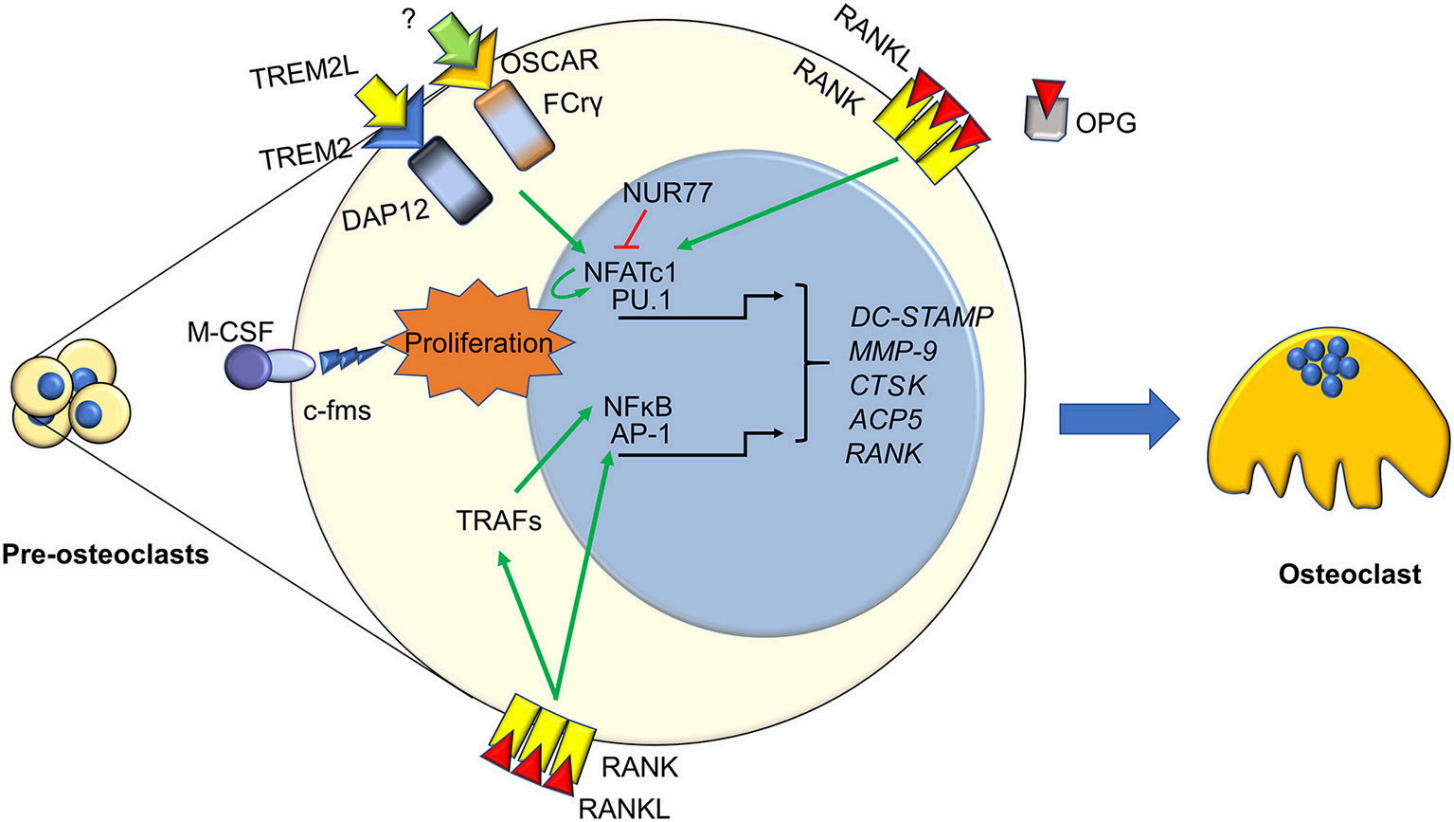
Immune factors in osteoclastogenesis. Osteoclasts differentiation from pre-osteoclasts involves several factors, most of which are derived from the immune system. Signaling from TREM2, OSCAR, c-FMS, and RANK cause the nuclear translocation of several transcription factors activating pre-osteoclasst proliferation and differentiation. These include the master osteoclastogenesis controllers NFATc1, which also self-amplifies, and NFkB, along with the early commitment factor PU.1 and AP1. This is enacted both directly by RANK, and indirectly through TRAFs and PLCγ. Osteoclastogenesis can be hindered by several factors, two key ones are the decoy receptor for RANKL, OPG, and the immune factor NUR77, which can inhibit NFATc1 stopping its self-amplifcation loop. The final outcome of these molecular pathways is the transcription of key osteoclast genes, such as DC-STAMP, MMP9. *CTSK*, ACP5, and RANK, which eventually results in the generation of a mature osteoclast.

In the past, bone was seen as a static tissue, a simple “scaffolding” for all the other organs. Now we know bone is actually extremely dynamic, undergoing continuous cycles of modeling during growth and remodeling during adulthood, which guarantee adequate mechanical properties and proper bone shape (7). Bone modeling and remodeling are guaranteed by the action of three types of bone cells: osteoclasts, which resorb bone, osteoblasts, which depose bone, and osteocytes, which are former osteoblasts buried in bone matrix, controlling bone mechano-physiology, and able to resorb and depose bone. The cycle of bone remodeling happens following these 4 phases: (1) latent phase: bone-lining cells are activated by osteocytes following a stimulus, starting osteoclast differentiation (Figure 1) and exposing the bone surface; (2) activation phase: osteoclasts resorb the portion of bone left exposed by the bone-lining cells. When they are done resorbing, they detach from bone and undergo apoptosis; (3) reverse phase: macrophage-like reverse cells migrate to the resorbed lacuna and clean it of the debris left by osteoclasts. Reverse cells also secrete factors that summon osteoblasts in the resorption lacuna; (4) formation phase: this is the longest phase in bone remodeling, lasting up to 6 months. Osteoblasts occupy the resorption lacuna and fill it up with organic osteoid matrix, which they then mineralize (7). In this last phase, osteoblasts may undergo apoptosis, or embed themselves in the bone matrix they produce, eventually becoming osteocytes (8). Bone modeling and remodeling are very similar as far as the mechanisms and the cellular players go. The key difference is that modeling happens during growth and fracture repair, and guarantees bone mass accrual, while remodeling happens in adulthood, does not change bone mass, but keeps mechanical properties at physiological levels, by continuously renewing the bone matrix. Although this is quite an accurate depiction of “normal” bone modeling/remodeling, it has become clear in the last years, that the molecular and cellular players involved in the maintenance and accrual of bone mass are many more than originally expected. A key player in the maintenance of bone mass in bone pathophysiology is undoubtedly the immune system, giving rise to an extremely important field of research: osteoimmunology. This will be discussed in the following paragraphs and chapters.

## Osteoimmunology

The term osteoimmunology was likely adopted for the first time by Arron and Choi (9), to describe the phenomenon of T-cell-mediated regulation of osteoclasts. Most of the research projects related to bone-immune system cross-talk are quite recent and mainly focused on the influence of the immune system on osteoclast physiology. As a matter of fact, these cells share a common origin with immune cells, since they both arise from bone marrow hematopoietic stem cells (10). Moreover, like other hematopoietic cells, osteoclast precursors can be detected as circulating cells in blood and their number increases under inflammatory conditions, characterized by high levels of the potent inflammatory cytokine Tumor Necrosis Factor (TNF)-α (11, 12).

### Bone Cells and the Immune System

In the following paragraphs, the many ways through which bone cells are linked to the immune system, and how they regulate immunity, will be discussed.

#### Osteoblasts

**Figure 2:**
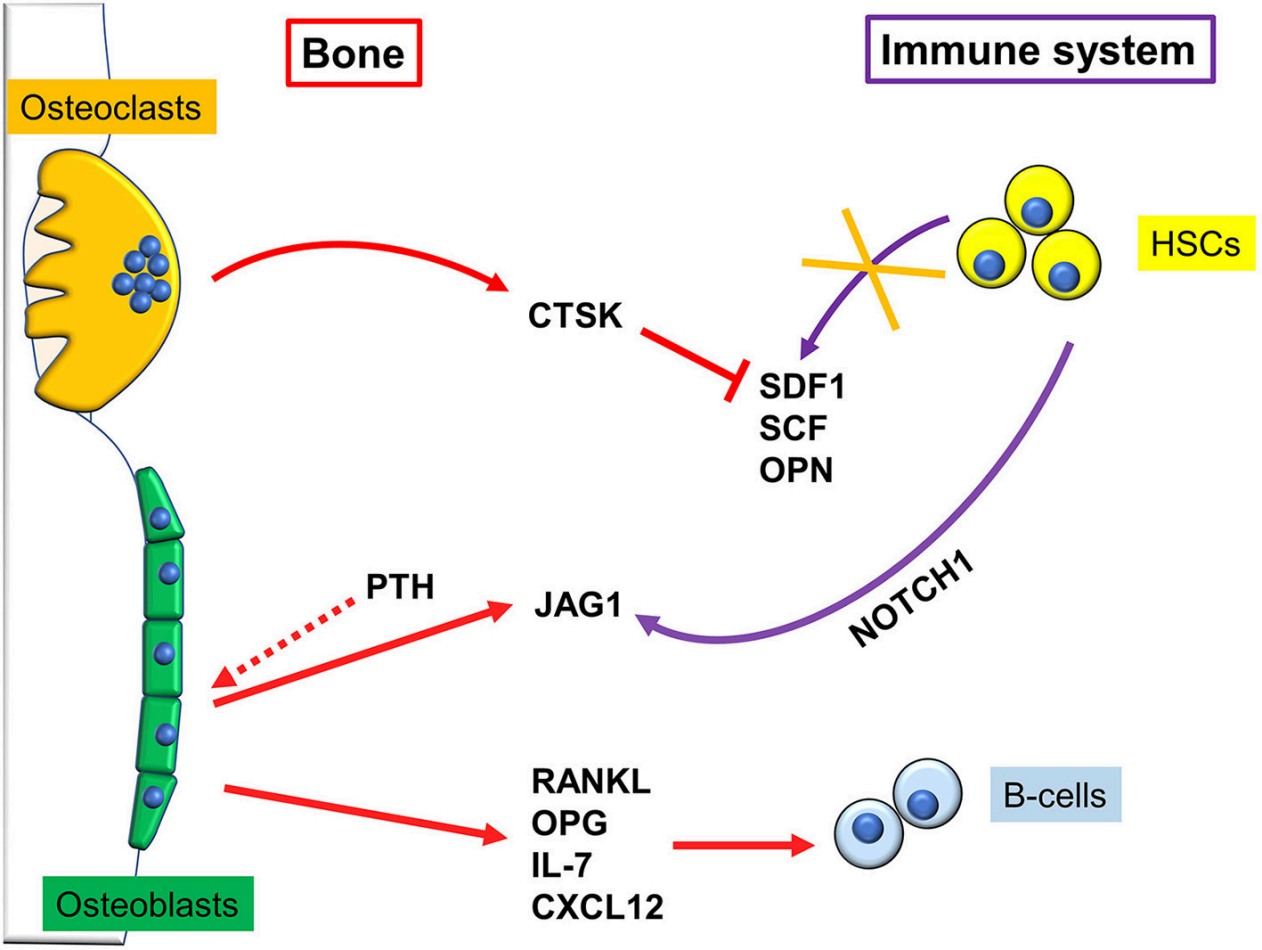
Regulation of immune cells by bone cells. Osteoclasts reduce hematopoietic stem cells (HSCs) homing by secreting cathepsin K (CTSK), which in turn degrades stromal cell-derived factor (SDF)1, stem cell factor (SCF), and osteopontin (OPN) depriving the bone niche of HSC-binding sites, which causes their mobilization. Osteoblasts, after stimulation with pro-osteoblastogenic factors such as intermittent parathyroid hormone (PTH), express Jagged1 (Jag1), which binds NOTCH1 on HSCs, and allows them to engraft and survive into the endosteal niche. B cells and bone cells communicate in multiple ways. For example, osteoblasts produce IL-7 and the chemokine CXCL12, that are fundamental for B-cells survival and activity.

A pivotal work demonstrating a close connection between bone and the hematopoietic compartment came in 2003, when Calvi and colleagues demonstrated that mice genetically engineered to express a constitutively active PTH/PTHrP receptor in osteoblasts, had more Hematopoietic Stem Cells (HSCs). This was due to an increase in osteoblastic Jagged1, which in turn mediated said effect through activation of Notch1 (Figure 2). Intriguingly, when Calvi and colleagues myeloablated WT mice, and subsequently performed bone marrow transplantation, treatment with intermitting doses of PTH improved the engraftment rate to 100%. This could be important for human conditions that require similar procedures (13). Likewise, Zhang et al. found a direct correlation between the number of a subset of osteoblasts, called Spindle-shaped-N-cadherin^+^CD45^−^ Osteoblasts (SNOs) and the number of HSCs. Moreover, long-term HSCs were found bound to SNOs by an N-cadherin/βcatenin-dependent mechanism (14). These data clearly demonstrate that a subpopulation of osteoblasts plays a crucial role in HSCs regulation, thus identifying in the bone marrow the so called “endosteal niche” besides the well-known vascular niche (15).

Later on, Zhu and colleagues definitely demonstrated that osteoblasts contribute to the commitment and differentiation of B lymphocytes from hematopoietic stem cells (16). In particular, in mice subjected to osteoblast conditional ablation, B-lymphocyte differentiation was compromised because of the lack of transition from Rag2^−^ to Rag2^+^ committed lymphoid progenitors. This effect was likely due to osteoblast secretion of InterLeukin (IL)-7 and C-X-C motif chemokine Ligand (CXCL)12 *alias* Stromal cell-Derived Factor (SDF) 1, two cytokines pivotal for B cells differentiation (17, 18). A collection of the main immune-derived factors promoting or hindering osteoblast differentiation and activity is present in Table 1.

**Table 1:** Factors produced by immune cells influencing osteoblast activity.

**Factor**	**Source**	**Action**	**References**
IL-11	Bone marrow stromal cells	Increase osteoblast activity	(19)
IL-6	Bone marrow stromal cells, osteoblasts, macrophages, muscle tissue, fibroblasts	Reduce osteoblast differentiation and activity	(20, 21)
IFN-γ	T-cells, NK-cells	Increase osteoblast activity	(22, 23)
IL-17F	Th17 cells	Increase osteoblast activity	(24, 25)
IL-15	PG-stimulated stromal cells, NK-cells	Reduce viability, increase apoptosis	(26)
OSM	B-cells	Increase osteoblast activity	(27)

#### Osteocytes

Osteocytes are the main producers of Receptor Activator of Nuclear factor Kappa B ligand (RANKL) in the bone, therefore since this cytokine is crucial not only for osteoclasts but also for lymphocyte development (see next paragraph) it is conceivable that this cell type could influence the immune system. Indeed, it has been demonstrated that RANKL arising from osteocytes contributes to the increased osteoclastogenesis and bone loss observed in estrogen deficient conditions. Moreover, specific deletion of the Rankl gene in osteocytes also prevented the increase in B cell formation caused by estrogen deficiency (28). In support of the relationship between osteocytes and the immune system, Sato and colleagues found that *in vivo* ablation of osteocytes leads to severe lymphopenia, caused by the loss of lymphoid- supporting stroma in the thymus and in the bone marrow, which is reverted by re-establishing the osteocyte population (29).

#### Osteoclasts

Osteoclasts have been shown to regulate the HSC niche both directly and indirectly through osteoblasts (Figure 2). Firstly, osteoclasts can increase HSC mobilization by secreting cathepsin K, a crucial protein for osteoclast function, which cleaves SDF1, OsteoPontiN (OPN), and Stem Cell Factor (SCF), depriving the bone niche of HSC-binding sites. Consequently, HSCs mobilize to the circulation and are no longer kept quiescent (30). Furthermore, it has been shown that *oc/oc* mice, which have an inactivating mutation in the *T Cell, Immune ReGulator 1, ATPase, H*^+^ *transporting, lysosomal V0 protein A3* (*Tcirg1)* gene and therefore almost entirely lack osteoclasts activity (31), have an overrepresented Mesenchymal Stem Cell (MSC) fraction. However, despite the higher number of precursors, MSCs differentiate less into osteoblasts, which impairs osteoblast-mediated HSCs homing to bone (32). Therefore, osteoclasts number and activity need to be tightly regulated, because any alteration could lead to excessive HSCs mobilization. Furthermore, *oc/oc* mice present with improper B lymphopoiesis, which is blocked at the pro-B stage, leading to fewer mature B-cells. T-cell activation is also affected, leading to a form of B-T-cells immunodeficiency (33).

As it is well-known, osteoclast differentiation strictly relies on the RANKL/RANK pathway (34, 35). RANKL interacts with its receptor RANK expressed by osteoclast precursors, thus recruiting TNFR-Associated Factors (TRAFs), which in turn trigger osteoclast differentiation by stimulating nuclear translocation of Nuclear Factor k-light-chain-enhancer of activated B cells (NFkB), Activator Protein 1 (AP1) complex and Nuclear Factor of Activated T-cells, cytoplasmic, calcineurin-dependent 1 (NFATc1) (36). All these factors stimulate transcription of several osteoclast-specific genes, such as Tartrate Resistant Acid Phosphatase (TRAcP), calcitonin receptor, cathepsin K, OSteoClast Associated Receptor (OSCAR), alpha V β3 integrin, Matrix Metalloproteinase (MMP) 9, and Dendritic Cell-Specific Transmembrane Protein (DC-STAMP) the latter involved in osteoclast fusion [Figure 1, (7)].

Of note, RANKL is also produced by activated T-lymphocytes as soluble form and is expressed in lymph nodes and thymus. Its importance in the immunological context was demonstrated by the fact that mice lacking RANKL showed not only a bone phenotype, resulting in osteopetrosis due to the lack of osteoclasts, but also presented with immunological defects, with impaired lymphocytes development and lack of lymph node organogenesis (37). Consistently, Dougall et al. demonstrated that RANK is essential for osteoclast and lymph node development, since RANK knockout mice showed an osteopetrotic phenotype along with a lack of peripheral lymph nodes and a marked deficiency in B and T lymphocytes (38). In contrast, OsteoProteGerin (OPG) is a decoy receptor for RANKL, belonging to the TNF Receptor (TNFR) superfamily, which prevents RANKL interaction with its receptor RANK, eventually leading to inhibition of osteoclast formation (35). Not only osteoblasts but also B lymphocytes produce OPG, thus concurring to regulate osteoclastogenesis (39).

The awareness of a close interconnection between bone and immune system was increased by Takayanagi et al. (40), who demonstrated that mice lacking Immunoreceptor Tyrosine-based Activation Motif (ITAM)-harboring adaptors, Fc Receptor common Gamma subunit (FcRγ) and DNAX-Activating Protein (DAP)12, presented with an osteopetrotic phenotype caused by a reduction of osteoclast differentiation. Indeed, they showed that RANKL/RANK pathway requires the ITAM-dependent costimulatory signals to activate osteoclast differentiation (5). Later, they found that FcRgamma and DAP12 associated to OSCAR and receptor expressed on myeloid cells 2 (TREM2), respectively, eventually leading to PhosphoLipase C (PLC)γ activation, which in turn activates calcium signaling, necessary for NFATc1 auto-amplification (41, 42). Osteoclasts also express Bruton's Tyrosine Kinase (Btk) and Tec, which have a physiological role in B cells (43), and mice double knock out for both these kinases manifest osteoclast-poor osteopetrosis, likely due to a suppression of the RANKL induced phosphorylation of PLCγ (44).

Regulation of osteoclast formation is a complex mechanism, calling into questions more and more pathways. One of the last is NR4A1 (alias Nur77), which belongs to the orphan nuclear receptor family, already known to be a key regulator of myeloid and lymphoid differentiation and function (45, 46). Recent findings demonstrated a role for this nuclear orphan receptor in the suppression of osteoclast differentiation (47). Moreover, Scholtysek and colleagues clarified a role for NR4A1 in controlling pre-osteoclast recruitment and migration, with an effect linked to the myeloid lineage. In fact, myeloid-specific but not osteoblast-specific deletion of NR4A1 resulted in osteopenia due to an increase of osteoclast number (48). The immune-related factors influencing osteoclast formation and biology are many more; a list of the most important ones is presented in Table 2.

**Table 2:** Secreted and membrane-bound immune factors promoting osteoclastogenesis.

**Factor**	**Source**	**References**
RANKL	Osteoblasts, osteocytes, neutrophils, sinoviocytes, T-cells	(28, 37, 49)
OPG	Osteoblasts, B-cells	(35, 50)
M-CSF	Osteoblasts, activated T-cells	(6, 51, 52)
TNFα	Activated leucocytes	(11, 12)
IL-1α,−1β	Activated leucocytes, osteoblasts, synoviocytes, endothelial cells	(53)
IL-7	Osteoblasts, bone marrow stromal cells, leucocytes	(53)
IL-8	Activated leucocytes	(53)
IL-11	Bone marrow stromal cells	(19)
IL-23	Dendritic cells, Th17 T-cells	(24, 54)
IL-34	Dendritic cells, Th17 T-cells, synoviocytes, osteoblasts	(24, 53)
Prostaglandins	Bone marrow and bone cells	(55)
SOFAT	T-cells	(56)
IL-6	Bone marrow stromal cells, osteoblasts, macrophages, muscle tissue, fibroblasts	(19–21, 57, 58)
IFN-γ	T-cells, NK-cells	(22, 23)
IL-17A	Th17 cells	(24, 59, 60)
IL-15	PG-stimulated stromal cells, NK-cells	(26)

### Immune Cells in Bone Physiology

As described, bone cells can influence the immune system, and employ several immune factors for their physiologic function. The opposite is also true: immune cells can influence bone health in many ways, as will be described in the following paragraphs.

#### T-Cells

T-cells are a key component of adaptive immunity. These small and relatively rare cells have a key role not only in immunity, but also in osteoimmunology. T-cells are not all created equal, and in this group, we can find cytotoxic CD8^+^ T-cells, CD4^+^ T-helpers, further subcategorized in Th1, Th2, Th17, and T-reg cells, the latter having an important role in preventing excessive or improper (e.g. self-directed) immune response (61). The links between T-cells and bone biology are numerous: essentially all the subtypes of T-cells are able to influence bone cells (mostly osteoclasts). However, a particularly important role for Th17 and T-reg cells is emerging. Th17 cells have been proposed to be the most osteoclastogenesis-inducing T-cells. They are characterized by the expression of a cytokine signature: IL-17A, IL-17F (hence the name), IL-22, IL-26, and IFN-γ (62). These cells can induce Macrophage Colony-Stimulating Factor (*M-CSF*) and *RANKL* expression in osteoblasts and stromal cells (6), produce RANKL and TNF-α, while parallelly increasing *RANK* expression in osteoclast precursors (63). These features make them potent osteoclastogenesis inducers, which have been already described as players in human bone diseases, such as RA (64) and multiple myeloma (65). With regards to T-reg cells, their role is clearly anti-osteoclastogenic, and it is enacted through a soluble factors-mediated mechanism, as well as a contact-mediated mechanism (24). In fact, co-culture experiments of whole Peripheral Blood Mononuclear Cells (PBMCs) or T-reg-depleted PBMCs resulted in higher osteoclast formation in the former (66), which seems to be dependent on TGF-β and IL-4, while the latter was found to be Cytotoxic T-Lymphocyte Antigen (CTLA)4-dependent (67). In the aforementioned reports, *in vitro* bone resorption was reduced up to 80% by the action of T-reg cells, which makes them potentially very important in autoimmune osteolysis-inducing disease such as RA. In fact, this has been proposed and demonstrated in mice (68), although human studies are still lacking to date.

#### Dendritic Cells

Dendritic Cells (DCs) are antigen-presenting cells entrusted with the important role of directing cell-mediated immunity toward the right targets, as quickly as possible and avoiding self-immunity (69). Their role in bone biology has in fact been historically thought as mostly indirect, through T-cells (70). Dendritic cells are in fact not only able to present antigens to T-cells, but also to regulate their activity and subtype balance through cytokine signaling (69, 71). However, another interesting concept that could be important for RA is that DCs can transdifferentiate into osteoclasts through M-CSF and RANKL stimulation, as if they were osteoclast precursors. Since DCs are numerous in and around the inflamed synovium in RA, they could well-contribute to the osteolytic disease in RA (72). However, DCs have not been further investigated in the last years in osteoimmunology in human studies.

#### Neutrophils

Neutrophils also play a role in bone biology, and particularly in inflammation-induced bone loss (73). In fact, neutrophils are usually the first cell type migrating to damage sites, including bone (74), and they can secrete many chemokines, cytokines and small molecules, which are able to act as immunomodulatory factors. For example, by CCL2 and CCL20 secretion, neutrophils are able to summon Th17 cells (73) which in turn cause bone loss, as discussed in the above paragraphs. However, absence of neutrophils can be even more damaging to bone tissue, as it eventually results in local IL-17-driven inflammatory bone loss (75). Remarkably, activated neutrophils express RANKL in the inflammatory site, and if that site is the synovium, they can actively participate in osteoclastogenesis, which increases RA-related osteolysis (76). In summary, although the role of neutrophils in osteoimmunology is not cut-and-dried, the general consensus is that activated neutrophils are osteoclastogenesis inducers, both directly and indirectly.

#### B-Cells

B-cell development relies on the production of several factors, including RANKL, OPG, IL-7, and CXCL12, which are produced by bone marrow stromal cells and osteoblasts [Figure 2, (50)]. As already mentioned, RANK knockout mice presented with a reduction in the number of mature B220^+^IgM^+^ and B220^+^IgG^+^ B-cells in lymph nodes (38, 77). Further studies demonstrated that not only RANKL arising from the bone marrow/bone compartment is crucial for B-cell development, but also B-cells themselves produce RANKL, which then acts as an autocrine factor (78). However, when RANK was conditionally deleted in the pro-B cells, B cell development was not affected (79) thus suggesting that RANKL could interact with an alternative receptor.

The evidence that B cells produce RANKL suggests that they could influence osteoclasts, and this is the case. In fact, Onal and colleagues demonstrated that mice lacking RANKL in B lymphocytes were partially protected from ovariectomy-mediated bone loss, through a mechanism counteracting the increase in osteoclasts number that is a hallmark of this mouse model. Conditional knock out of RANKL in T-lymphocytes had no effect on ovariectomy-induced bone loss (78). Interestingly, IL-7 transgenic mice showed focal osteolysis, besides the expected increase of pro-B and pre-B cells, while mice lacking the IL-7 receptor showed suppression in B-lymphocyte development associated to an increased bone mass (80).

*In vitro* studies have shown that purified B cells can be driven to differentiate into osteoclasts when treated with RANKL, thus acting as a source of osteoclast progenitors in *vitro* (81, 82). In contrast, *in vivo* lineage-tracing studies to investigate whether cells committed to the B cell lineage can act as osteoclast progenitors found that this was not the case, therefore the authors conclude that the role of B cells is not to act as osteoclast progenitors but as cells supporting osteoclasts formation (28).

#### Natural Killer (NK) Cells

Natural Killer (NK) cells, as all other lymphocytes, play a role in the regulation of bone homeostasis. In fact, several reports emerged stating that they are particularly important in bone destruction induced by RA, and they are also able to induce osteoblast cell death (26, 83, 84). This makes NK cells a potential therapeutic target to reduce RA-induced bone destruction. However, NK cells have also been described as helpful and necessary to slow down RA by recent reports (85), casting doubts over the efficacy of a hypothetical anti-NK-cells treatment in RA.

#### Osteomacs and Bone Marrow Macrophages

As other organs in the body, bone and bone marrow present with resident macrophages, which include bone marrow macrophages and osteal macrophages (86). The latter, also known as osteomacs, are F4/80 positive and TRAcP negative, and are located close to the bone surface. It has been recently demonstrated that in 2-day-old mouse calvarial osteoblasts a small population of CD45^+^F4/80^+^ osteomacs can be detected (87). This subpopulation cooperates with osteoblasts and megakaryocytes to promote hematopoietic progenitor and HSC function (87, 88). The same authors also demonstrated that highly purified CD45^+^F4/80^+^ osteomacs from neonatal calvarial osteoblasts can differentiate into TRAcP positive osteoclasts able to resorb bone (88).

Notably, *in vitro* and *in vivo* studies demonstrated a role for these bone macrophages in osteoblast differentiation by producing Bone Morphogenetic Proteins (BMPs) (89) and Oncostatin M (27). Moreover, Chang and colleagues found that depletion of osteal macrophages in primary osteoblasts inhibited their differentiation (90). *In vivo* approaches able to selectively ablate osteal macrophages but not osteoclasts showed that their lack determined a decrease in bone formation, a reduction in bone growth in young mice and osteoporosis (90–92). Therefore, osteomacs are versatile cells, able to regulate bone mass, become osteoclasts, and actively participate in the homeostasis of the immune system.

#### Inflammation and Inflammatory Factors

Several cells of the immune system (T and B cells, NK cells, monocyte/macrophage and dendritic cells) produce InterFeroN (IFN)-γ, which has a pivotal role in innate and adaptive immune responses as well as in the regulation of inflammation (22, 23). In bone, IFN-γ affects both osteoblasts and osteoclasts. The former, which produce low levels of this cytokine, are indeed positively affected, since IFN-γ increases osteoblast differentiating genes, like Runt related transcription factor 2 (Runx2), Osterix, ALkaline Phosphatase (ALP) and Osteocalcin (93, 94) and mice knock out for the IFN-γ receptor showed reduced osteoblast differentiation (95). Conversely, IFN-γ has an inhibitory effect on adipogenesis (96).

Much data has been collected about the inhibitory effect of IFN-γ on osteoclast differentiation [for a review see (23)]. This protein is known to counteract the effect of M-CSF on osteoclast precursors by reducing the expression of its receptor c-fms, eventually leading to a reduced pool of Rank-positive preosteoclasts (97). Furthermore, IFN-γ promotes TRAF6 degradation (53, 98), thus inhibiting the downstream signaling which involves JNK and NFkB, while it induces osteoclast apoptosis by activating Fas-FasL-mediated death signaling (99). However, other studies show a pro-osteoclastogenic effect of IFN-γ in the late state of differentiation. This depends on the upregulation of NFATc1 and c-fos, which in turn stimulate *DC-STAMP* expression, thus promoting osteoclast fusion (100). Another indirect pro-osteoclastogenic effect of IFN-γ is due to its ability to increase the secretion of CXCL10, also known as Interferon gamma induced Protein 10 (IP-10), by macrophages and this cytokine in turn stimulates the production of RANKL and TNF-α by T cells (101).

Inflammatory diseases, such as RA, periodontal disease (see later), systemic lupus erythematous, inflammatory bowel diseases and cystic fibrosis are all characterized by bone loss (6, 102–106), which is not only secondary to the employ of anti-inflammatory corticosteroid therapies (107), but is also due to a direct effect of inflammatory cytokines on osteoclasts, thus creating a sort of “vicious cycle”.

So far, a plethora of inflammatory cytokines have been identified as positive modulators of osteoclasts [for a review see (53)]. Above all the proinflammatory cytokine TNF-α, which directly stimulates osteoclastogenesis by a mechanism independent of RANKL (108) as well as indirectly, by promoting RANK expression on preosteoclasts (109) and increasing RANKL and M-CSF production by osteoblasts and activated T cells (50, 51).

The IL-6 family includes at least three pro-osteoclastogenic cytokines: IL-6, -11 and -23. The first stimulates osteoclastogenesis by a mechanism independent of RANKL, since the presence of OPG does not blunt this effect (57). This is accompanied by a positive stimulation of RANKL production by stromal cells and osteoblasts (19, 58). As for osteoblasts, IL-6 reduces their differentiation *in vivo* (20) and *in vitro* by affecting the MEK2 and Akt2 pathways (21).

Also, IL-11 has a pro-osteoclastogenic effect (19), while on the osteoblast side it seems to be pro-osteogenic (110, 111).

IL-23 is mainly produced by dendritic cells and macrophages, and it indirectly stimulates osteoclastogenesis by increasing RANK and RANKL expression by osteoclast precursors and osteoblasts, respectively (54). Other factors strongly promoting osteoclastogenesis are prostaglandins (55), IL-1α, -1β, -7, -8, and -34 (53).

## Osteoimmunology in Bone and Immune Diseases

As it is easy to imagine, the link between bone and the immune system in physiology is also maintained in pathological conditions: many diseases affecting bone have an immunologic origin, while some immunological disorders, such as acute myeloid leukemia, can originate from bone-derived signals, as will be described in the following paragraphs.

### Rheumatoid Arthritis

Rheumatoid arthritis (RA) is a T helper (Th) type 1, degenerative disease with strong genetic predisposition factors (up to 50%), characterized by synovitis, persistent inflammation and the generation of antibodies against endogenous proteins, especially rheumatoid factor (112). Takayanagi's group demonstrated a close connection between RA and osteoclast deregulation (6). Indeed, despite the high levels of IFN-γ, which is known to inhibit osteoclastogenesis, the T-cells' activation observed in this pathology results in an exacerbated osteoclast activation. This is due to the action of a subset of activated T-cells, called Th-17 because they produce IL-17. Of note, in a mouse model of RA, IL-17 ablation reduced bone destruction (59). This cytokine is of interest, because it stimulates the expression of more pro-osteoclastogenic cytokines, including IL-6, IL-8, and TNF-α also in absence of RANKL (113, 114). Moreover, in the synovium of RA patients, IL-17 induces the production of IL-32, which in turn stimulates IL-17 expression (60) creating a feed-forward loop. Therefore, this cytokine plays a crucial role not only in the onset of RA but also in the bone destruction that this disease presents with (6). Furthermore, IL-6 production increases RANKL expression in synoviocytes, leading to further exacerbated osteoclastic bone destruction (49).

### Osteoporosis

Estrogen deficiency is the leading cause of osteoporosis in post-menopausal women. Pacifici and colleagues found that this condition results in increased production of inflammatory cytokines. This was a cornerstone discovery, that elucidated a new facet of post-menopausal osteoporosis, describing it as an inflammatory disease (115). The protective effect of estrogen in bone has been well characterized and is mainly due to a direct action on both osteoclasts and osteoblasts. In the former, estrogens significantly increase apoptosis (116, 117) and reduce RANKL-dependent osteoclast formation (118), while in osteoblasts they exert an anabolic effect by at least increasing osteoblast survival and collagen I production (119). Consistently, it has been demonstrated that estrogens suppress RANKL production not only in osteoblasts but also in T and B cells (120), while the lack of estrogens increases the release of pro-osteoclastogenic cytokines (i.e., TNF-α and RANKL) by activated T cells (121–123) thus indirectly identifying immune cells as additional players in the onset of osteoporosis. Estrogens withdrawal also leads to a significant increase of B-cells' number (124).

Regarding the role of immune cells-derived-IFN-γ in osteoporosis, results are conflicting (23). In particular, while Breuil and colleagues found reduced secreted levels of IFN-γ by CD4^+^ T-cells in osteoporotic patients (121) previous studies did not find any differences (125, 126).

### Periodontal Disease

Another pathological phenomenon in which immune cells deregulation causes bone loss is periodontal disease, where activated B and T cells concur to stimulate osteoclast resorption by producing RANKL (127, 128). Consistently, Weitzmann and colleagues identified a T cell-secreted cytokine, named Secreted Osteoclastogenic Factor of Activated T-cells (SOFAT) that promotes osteoclastogenesis independently of RANKL (129, 130). Moreover, increased mRNA expression of SOFAT has been found in human periodontal samples, while SOFAT injection induced osteoclast formation in a mouse model of periodontal disease (56).

### Bone Fracture Repair

This physiological process is orchestrated not only by bone cells, but also by immune cells, whose deregulation can delay fracture repair (131, 132). In fact, in conditions of B and T cells depletion, an impairment of bone regeneration, due to a reduction of osteoblast differentiation and bone mineralization, has been observed (25, 133). Consistently, immunocompromised HIV patients present with a delay of fracture healing repair (131).

One of the earliest phases of bone healing is characterized by an inflammatory state, with the release of IL-1, IL-6, and TNF-α, which recruit B and T lymphocytes, the latter having a pro-osteogenic role by releasing IL-17F (25). In fact, at variance with IL-17A, known for its pro-osteoclastogenic role, the pro-inflammatory cytokine IL-17F is expressed during bone healing, where it increases Col1a1, osteocalcin, and bone sialoproteins in treated osteoblasts (25). Neutrophil granulocytes are also present during the early phase of bone repair, and they clean debris and damaged cells in the site of injury (134). Another crucial step for bone fracture repair is the recruitment of MSCs which again involves immune cells (i.e., NK cells and macrophages) since they produce chemoattractant molecules such as CXCL7 (alias NAP2) and Monocyte Inflammatory Protein (MIP)-1α (135, 136). Once recruited, MSCs are fostered to differentiate toward the osteoblast lineage, through macrophage-derived BMPs (137) while activated monocytes stimulate the expression of Runx2 (138). At the same time, once the bone-repair process activates, the inflammatory reaction should be switched off in order to avoid any inflammation-derived damage. To this aim, MSCs exert an immunosuppressive role, by stimulating the differentiation of T-reg lymphocytes, inducing the apoptosis of the pro-inflammatory Th1 and Th17 lymphocytes and inhibiting migration of B-lymphocytes (139–142).

### Myelodysplasia and Acute Myeloid Leukemia

Another strong link between bone and the immune system is the fact that osteoblasts can influence the progression of pre-neoplastic and neoplastic transformations in the myeloid lineage. In fact, osteoblasts are able to slow down leukemia progression in mouse, creating an unfavorable microenvironment for leukemic blast growth (143). Consistently, osteoblast number is reduced by more than half in leukemic patients. Simulating this situation by mouse genetics, causes leukemic blasts to grow faster and engraft better (143). The same authors demonstrated that osteoblasts have another tight link to human leukemia: osteoblasts that have been genetically engineered to express a constitutively active form of β-catenin, are able to induce leukemic transformation in myeloid cells, causing MyeloDysplaSia (MDS) and then Acute Myeloid Leukemia (AML) (144).

The concept of bone cells inducing malignant transformation, however, was not new, since already a few years earlier, Raaijmakers and colleagues found that ablating the miRNA processing protein dicer from osteoblast progenitors induces dysfunctional haematopoiesis, eventually leading to MDS and AML development (145). The field of “niche-induced leukemia” has received much attention, and still many groups are working on this topic to date.

## Latest Developments in Osteoimmunology

In the last few years, despite much of the field has already emerged, several groups are still actively discovering new molecules that can be considered part of the osteoimmunology world. This has been the case for a secreted protein named homologous to Lymphotoxin, exhibits Inducible expression and competes with HSV Glycoprotein D for binding to Herpesvirus entry mediator, a receptor expressed on T lymphocytes (LIGHT, a.k.a. Tumor Necrosis Factor SuperFamily member 14, TNFSF14), which has been linked to increased bone resorption in osteoarthritis more than 10 years ago (146, 147), and has known a *renaissance* in the last few years as target for bone loss (148) and biomarker for bone disease in multiple myeloma (149). This molecule seems to have a dual effect in bone: high levels are linked to bone loss, and so is its absence. The mechanisms involving it are therefore quite complex, although agonists and antagonists of the LIGHT pathway are in development and testing. This behavior is also common to another regulator of bone mass that has recently emerged in the last few years: LipoCaliN-2 (Lcn2). This protein is also called Neutrophil Gelatinase-Associated Lipocalin (NGAL), since it can bind and stabilize MMP9, a crucial factor for neutrophil extravasation. Furthermore, Lcn2 is also readily overexpressed during inflammation, and following treatment with TNFα, IL-17, and IL-1β, and its role in inflammatory diseases is only starting to emerge; what is sure is that this molecule can be considered a player in innate immunity. In 2009, we discovered that Lcn2 is strongly overexpressed in osteoblasts following *in vitro* mechanical unloading (150). Furthermore, we confirmed these findings *in vivo*, which led to the concept that Lcn2 is a mechanoresponsive gene that regulates bone homeostasis (151). Surprisingly though, removing this protein genetically, reduces bone mass instead of increasing it (152). This is likely due to the fact that Lcn2 impairs osteoblasts when overexpressed, and impairs energy metabolism when removed, which causes an indirect osteoblast dysfunction (152). The role of Lcn2 in bone is still under investigation by ours and other groups where it has been found to influence hematopoiesis (153), and even appetite through the melatonin receptor MC4R (154).

## Conclusions

The concept of osteoimmunology is aging well, almost 20 years since the term was coined. This way of interpreting bone and the immune system has been steadily providing new insights about how the two of them operate and cooperate. As an example, the role of pro-inflammatory cytokines in promoting osteoclastogenesis, and the many parallelisms between immune cells and osteoclasts have proved crucial to understand the biology of these giant bone-eating cells. Intriguingly, the control mechanisms between bone and the immune system are complex, tightly interconnected, and involve many players. The underlying complexity of this field has made it difficult for researchers to find clear-cut results, the kind that leads to the direct clinical application. Nevertheless, thanks to the effort of many scientists, nowadays clinics can use drugs, classically employed to treat osteoporosis, for immunological diseases [e.g., Denosumab for RA; (155)]. In conclusion, although the study of osteoimmunology has provided many answers, it also raised more questions, which we need to answer in order to improve standards of care for patients of both immune and bone disorders, by exploiting the cross-talk between these two remarkable systems, which are actually starting to look like a single one.

## Author Contributions

MP prepared the cartoons and the tables. NR drafted the manuscript, MP and NR finalized, reviewed and approved the manuscript in its final form.

### Conflict of Interest Statement

The authors declare that the research was conducted in the absence of any commercial or financial relationships that could be construed as a potential conflict of interest.

## References

[1] Fernandez-Real JM Izquiredo M Ortega F Gorostiaga E Gomez-Ambrosi J Moreno-Navarrete JM. The relationship of serum osteocalcin concentration to insulin secretion, sensitivity and disposal with hypocaloric diet and resistance training. J Clin Endocrinol Metab. (2009) 94:237–45. 10.1210/jc.2008-0270 18854399

[2] Hwang YC Jeong IK Ahn KJ Chung HY. The uncarboxylated form of osteocalcin is associated with improved glucose tolerance and enhanced beta-cell function in middle-age male subjects. Diabetes Met Res Rev. (2009) 25:768–72. 10.1002/dmrr.1045 19877133

[3] Yeap BB Chubb SA Flicker L McCul KA Ebeling PR Beilby JP. Reduced serum total osteocalcin is associate with metabolic syndrome in older men via waist circumference, hyperglycemia, and triglyceride levels. Eur J Endocrinol. (2010) 163:265–72. 10.1530/EJE-10-0414 20501596

[4] Wei J Karsenty G. An overview of the metabolic functions of osteocalcin. Curr Osteopor Rep. (2015) 13:180–5. 10.1007/s11914-015-0267-y 25809656

[5] Koga T Inui M Inoue K Kim S Suematsu A Kobayashi E. Costimulatory signals mediated by ITAM motif cooperate with RANKL for bone homeostasis. Nature. (2004) 428:758–63. 10.1038/nature02444 15085135

[6] Sato K Suematsu A Okamoto K Yamaguchi A Morishita Y Kadono Y. Th17 functions as an osteoclastogenic helper T cell subset that links T cell activation and bone destruction. J Exp Med. (2006) 203:2673–82. 10.1084/jem.20061775 17088434 PMC2118166

[7] Cappariello A Maurizi A Veeriah V Teti A. The great beauty of osteoclast. Arch Biochem Biophys. (2014) 561:13–21. 10.1016/j.abb.2014.06.017 25282390

[8] Capulli M Paone R Rucci N. Osteoblast and osteocyte: games without frontiers. Arch Biochem Biophys. (2014) 561:3–12. 10.1016/j.abb.2014.05.003 24832390

[9] Arron JR Choi Y. Bone versus immune system. Nature. (2000) 408:535–6. 10.1038/35046196 11117729

[10] Walker DG. Bone resorption restored in osteopetrotic mice by transplants of normal bone marrow and spleen cells. Science. (1975) 190:784–5. 10.1126/science.1105786 1105786

[11] Li P Schwarz EM O'Keefe RJ Ma L Looney RJ Ritchlin CT. Systemic tumor necrosis factor alpha mediates an increase in peripheral CD11bhigh osteoclast precursors in tumor necrosis factor alpha-transgenic mice. Arthritis Rheum. (2004) 50:265–76. 10.1002/art.11419 14730625

[12] Yao Z Li P Zhang Q Schwarz EM Keng P Arbini A. Tumor necrosis factor-alpha increases circulating osteoclast precursor numbers by promoting their proliferation and differentiation in the bone marrow through up-regulation of c-Fms expression. J Biol Chem. (2006) 281:11846–55. 10.1074/jbc.M512624200 16461346

[13] Calvi LM Adams GB Weibrecht KW Weber JM Olson DP Knight MC. Osteoblastic cells regulate the haematopoietic stem cell niche. Nature. (2003) 425:841–6. 10.1038/nature02040 14574413

[14] Zhang J Niu C Ye L Huang H He X Tong WG Identification of the hematopoietic stem cell niche and control of the niche size. Nature. (2003) 425:836–41. 10.1038/nature02041 14574412

[15] Zhu J Emerson SG. A new bone to pick: osteoblasts and the haematopoitic stem-cell niche. Bioessays. (2004) 26:595–9. 10.1002/bies.20052 15170855

[16] Zhu J Garrett R Jung Y Kim N Wang J Joe GJ. Osteoblasts support B-lymphocyte commitment and differentiation from hematopoietic stem cells. Blood. (2007) 109:3706–12. 10.1182/blood-2006-08-041384 17227831

[17] Egawa T Kawabata K Kawamoto H Amada K Okamoto R Fujii N. The earliest stages of B cell development require chemokine stromal cell-derived factor/pre-B cell growth- stimulating factor. Immunity. (2001) 15:323–34. 10.1016/S1074-7613(01)00185-6 11520466

[18] Miller JP Izon D DeMuth W Gerstein R Bhandoola A Allman D. The earliest step in B lineage differentiation from common lymphoid progenitors is critically dependent upon interleukin 7. J Exp Med. (2002) 196:705–11. 10.1084/jem.20020784 12208884 PMC2193997

[19] Girasole G Passeri G Jilka RL Manolagas SC. Interleukin 11: a new cytokine critical for osteoclast development. J Clin Invest. (1994) 93:1516–24. 10.1172/JCI117130 8163655 PMC294166

[20] De Benedetti F Rucci N Del Fattore A Peruzzi B Paro R Longo M. Impaired skeletal development in interleukin-6-transgenic mice: a model for the impact of chronic inflammation on the growing skeletal system. Arthritis Rheum. (2006) 54:3551–63. 10.1002/art.22175 17075861

[21] Kaneshiro S Ebina K Shi K Higuchi C Hirao M Okamoto M. IL-6 negatively regulates osteoblast differentiation through the SHP2/MEK2 and SHP2/Akt2 pathways *in vitro*. J Bone Miner Metab. (2014) 32:378–92. 10.1007/s00774-013-0514-1 24122251

[22] Schroder K Hertzog PJ Ravasi T Hume DA. Interferon-gamma: an overview of signals, mechanisms and functions. J Leukoc Biol. (2004) 75:163–89. 10.1189/jlb.0603252 14525967

[23] Tang M Tian L Luo G Yu X. Interferon-gamma-mediated osteoimmunology. Front Immunol. 9:13. 10.3389/fimmu.2018.01508 30008722 PMC6033972

[24] Mori G D'Amelio P Faccio R Brunetti G. The Interplay between the bone and the immune system. Clin Dev Immunol. 2013:720504. 10.1155/2013/720504 23935650 PMC3725924

[25] Nam D Mau E Wang Y Wright D Silkstone D Whetstone H. T-lymphocytes enable osteoblast maturation via IL-17F during early phase of fracture repair. PLoS ONE. (2012) 7:e40044. 10.1371/journal.pone.0040044 22768215 PMC3386936

[26] Takeda H Kikuchi T Soboku K Okabe I Mizutani H Mitani A. Effect of IL-15 and natural killer cells on osteoclasts and osteoblasts in a mouse coculture. Inflammation. (2014) 37:657–69. 10.1007/s10753-013-9782-0 24287823

[27] Guihard P Boutet MA Brounais B David E Brion R Delecrin J. Induction of osteogenesis in mesenchymal stem cells by activated monocytes/macrophages depends on oncostatin M signaling. Stem Cells. (2012) 30:762–72. 10.1002/stem.1040 22267310

[28] Fujiwara Y Piemontese M Liu Y Thostenson JD Xiong J O'Brien CA RANKL (Receptor Activator of NFkB Ligand) produced by osteocytes is required for the increase in B cells and bone loss caused by estrogen deficiency in mice. J Biol Chem. (2016) 291:24838–50. 10.1074/jbc.M116.742452 27733688 PMC5122756

[29] Sato M Asada N Kawano Y Wakahashi K Minagawa K Kawano H. Osteocytes regulate primary lymphoid organs and fat metabolism. Cell Metab. (2013) 18:749–58. 10.1016/j.cmet.2013.09.014 24140021

[30] Kollet O Dar A Shivtiel S Kalinkovich A Lapid K Sztainberg Y. Osteoclasts degrade endosteal components and promote mobilization of hematopoietic progenitor cells. Nat Med. (2006) 12:657–64. 10.1038/nm1417 16715089

[31] Seifert MF Marks SC Jr. Morphological evidence of reduced bone resorption in the osteosclerotic (oc) mouse. Am J Anat. (1985) 172:141–53. 10.1002/aja.1001720204 3976544

[32] Mansour A Abou-Ezzi G Sitnicka E Jacobsen SE Wakkach A Blin-Wakkach C. Osteoclasts promote the formation of hematopoietic stem cell niches in the bone marrow. J Exp Med. (2012) 209:537–49. 10.1084/jem.20110994 22351931 PMC3302238

[33] Blin-Wakkach C Wakkach A Sexton PM Rochet N Carle GF. Hematological defects in the oc/oc mouse, a model of infantile malignant osteopetrosis. Leukemia. (2004) 18:1505–11. 10.1038/sj.leu.2403449 15284856

[34] Lacey DL Timms E Tan HL Kelley MJ Dunstan CR Burgess T. Osteoprotegerin ligand is a cytokine that regulates osteoclast differentiation and activation. Cell. (1998) 93:165–76. 10.1016/S0092-8674(00)81569-X 9568710

[35] Simonet WS Lacey DL Dunstan CR Kelley M Chang MS Luthy R Osteoprotegerin: a novl secreted protein involved in the regulation of bone density. Cell. (1997) 89:309–19. 10.1016/S0092-8674(00)80209-3 9108485

[36] Feng X Teitelbaum SL. Osteoclasts: new insights. Bone Res. (2013) 1:11–26. 10.4248/BR201301003 26273491 PMC4472093

[37] Yasuda H Shima N Nakagawa N Yamaguchi K Kinosaki M Mochizuki S. Osteoclast differentiation factor is a ligand for osteoprotegerin/osteoclastogenesis-inhibitory factor and is identical to TRANCE/RANKL. Proc Natl Acad Sci USA. (1998) 95:3597–3602. 10.1073/pnas.95.7.3597 9520411 PMC19881

[38] Dougall WC Glaccum M Charrier K Rohrbach K Brasel K De Smedt T. RANK is essential for osteoclast and lymph node development. Genes Dev. (1999) 13:2412–24. 10.1101/gad.13.18.2412 10500098 PMC317030

[39] Boyce BF Yao Z Xing L. Osteoclasts have multiple roles in bone in addition to bone resorption. Crit Rev Eukaryot Gene Expr. (2009) 19:171–80. 10.1615/CritRevEukarGeneExpr.v19.i3.10 19883363 PMC2856465

[40] Takayanagi H. Osteoimmunology: shared mechanisms and crosstalk between the immune and bone systems. Nature Rev Immunol. (2007) 7:292–304. 10.1038/nri2062 17380158

[41] Barrow AD Raynal N Andersen TL Slatter DA BIhan D Pugh N OSCAR is a collagen receptor that costimulates osteoclastogenesis in DAP12-deficient human and mice. J Clin Invest. (2011) 121:3505–16. 10.1172/JCI45913 21841309 PMC3163954

[42] Otero K Shinohara M Zhao H Cella M Gilfillan S Colucci A. TREM2 and β-catenin regulate bone homeostasis by controlling the rate of osteoclastogenesis. J Immunol. (2012) 188:2612–21. 10.4049/jimmunol.1102836 22312126 PMC3732181

[43] Ellmeier W Jung S Sunshine MJ Hatam F Xu Y Baltimore D. Severe B cell deficiency in mice lacking the tec kinase family members Tec and Btk. J Exp Med. (2000) 192:1611–24. 10.1084/jem.192.11.1611 11104803 PMC2193106

[44] Shinohara M Koga T Okamoto K Sakaguchi S Arai K Yasuda H. Tyrosine kinases Btk and Tec regulate osteoclast differentiation by linking RANK and ITAM signals. Cell. (2008) 132:794–806. 10.1016/j.cell.2007.12.037 18329366

[45] Hanna RN Carlin LM Hubbelin HG Nackiewicz D Green AM Punt JA. The transcription factor NR4A1 (Nur77) controls bone marrow differentiation and the survival of Ly6C-monocytes. Nat Immunol. (2011) 12:778–85. 10.1038/ni.2063 21725321 PMC3324395

[46] Tacke R Hilgendorf I Garner H Waterborg C Park K Nowyhed H. The transcription factor NR4A1 is essential for the development of a novel macrophage subset in the thymus. Sci Rep. (2015) 5:10055. 10.1038/srep10055 26091486 PMC4473761

[47] Li X Wei W Huynh H Zuo H Wang X Wang Y. Nur77 prevents excessive osteoclastogenesis by inducing ubiquitin ligase Cbl-b to mediate NFATc1. Elife. 4:e07217. 10.7554/eLife.07217 26173181 PMC4518709

[48] Scholtysek C Ipseiz N Böhm C Krishnacoumar B Stenzel M Czerwinski T. NR4A1 regulates motility of osteoclast precursors and serves as target for the modulation of systemic bone turnover. J Bone Miner Res. (2018) 33:2035–47. 10.1002/jbmr.3533 29949664

[49] Hashizume M Hayakawa N Mihara M. IL-6 trans-signalling directly induces RANKL on fibroblast-like synovial cells and is involved in RANKL induction by TNF-alpha and IL-17. Rheumatology (Oxford). (2008) 47:1635–40. 10.1093/rheumatology/ken363 18786965

[50] Nagasawa T. Microenvironmental niches in the bone marrow required for B-cell development. Nat Rev Immunol. (2006) 6:107–16. 10.1038/nri1780 16491135

[51] Kitaura H Zhou P Kim HJ Novack DV Ross FP Teitelbaum SL. M-CSF mediates TNF-induced inflammatory osteolysis. J Clin Invest. (2005) 115:3418–27. 10.1172/JCI26132 16294221 PMC1283943

[52] Weitzmann MN Cenci S Rifas L Brown C Pacifici R. Interleukin-7 stimulates osteoclast formation by up-regulating the T-cell production of soluble osteoclastogenic cytokines. Blood. (2000) 96:1873–8. 10961889

[53] Amarasekara DS Yun H Kim S Lee N Kim H Rho J. Regulation of osteoclast differentiation by cytokine networks. Immune Netw. 18:e8. 10.4110/in.2018.18.e8 29503739 PMC5833125

[54] Chen L Wei XQ Evans B Jiang W Aeschlimann D. IL-23 promotes osteoclast formation by up-regulation of receptor activator of NF-kappaB (RANK) expression in myeloid precursor cells. Eur J Immunol. (2008) 38:2845–54. 10.1002/eji.200838192 18958885

[55] Raisz LG. Prostaglandins and bone: physiology and pathophysiology. Osteoarthritis Cartilage. (1999) 7:419–21. 10.1053/joca.1998.0230 10419786

[56] Jarry CR Durante PM Freitas FF de Macedo CG Clemente-Napimoga JT Saba-Chujfi E. Secreted osteoclastogenic factor of activated T cells (SOFAT), a novel osteoclast activator, in chronic periodontitis. Hum Immunol. (2013) 74:861866. 10.1016/j.humimm.2013.04.013 23619471

[57] Kudo O Sabokbar A Pocock A Itonaga I Fujikawa Y Athanasou NA. Interleukin-6 and interleukin-11 support human osteoclast formation by a RANKL-independent mechanism. Bone. (2003) 32:1–7. 10.1016/S8756-3282(02)00915-8 12584029

[58] Yoshitake F Itoh S Narita H Ishihara K Ebisu S. Interleukin-6 directly inhibits osteoclast differentiation by suppressing receptor activator of NF-kappaB signaling pathways. J Biol Chem. (2008) 283:11535–40. 10.1074/jbc.M607999200 18296709

[59] Lubberts E Koenders MI van der Berg WB. The role of T-cell interleukin-17 in conducting destructive arthritis: lessons from animal models. Arthritis Res Ther. (2005) 7:29–37. 10.1186/ar1478 15642151 PMC1064899

[60] Moon YM Yoon BY Her YM Oh HJ Lee JS Kim KW. IL-32 and IL-17 interact and have the potential to aggravate osteoclastogenesis in rheumatoid arthritis. Arthritis Res Ther. (2012) 14:R246. 10.1186/ar4089 23148681 PMC3674587

[61] Danks L Takayanagi H. Immunology and bone. J Biochem. (2013) 154:29–39. 10.1093/jb/mvt049 23750028

[62] Adamopoulos IE Bowman EP. Immune regulation of bone loss by Th17 cells. Arthritis Res Ther. 10:225. 10.1186/ar2502 18983698 PMC2592787

[63] Adamopoulos IE Chao CC Geissler R Laface D Blumenschein W Iwakura Y. Interleukin-17A upregulates receptor activator of NF-kappaB on osteoclast precursors. Arthritis Res Ther. (2010) 12:R29. 10.1186/ar2936 20167120 PMC2875663

[64] Maddur MS Miossec P Kaveri SV Bayry J. Th17 cells: biology, pathogenesis of autoimmune and inflammatory diseases, and therapeutic strategies. Am J Pathol. (2012) 181:8–18. 10.1016/j.ajpath.2012.03.044 22640807

[65] Noonan K Marchionni L Anderson J Pardoll D Roodman GD Borrello I. A novel role of IL-17-producing lymphocytes in mediating lytic bone disease in multiple myeloma. Blood. (2010) 116:3554–63. 10.1182/blood-2010-05-283895 20664052 PMC4017298

[66] Kim YG Lee CK Nah SS Mun SH Yoo B Moon HB. Human CD4+CD25+ regulatory T cells inhibit the differentiation of osteoclasts from peripheral blood mononuclear cells. Biochem Biophys Res Commun. (2007) 357:1046–52. 10.1016/j.bbrc.2007.04.042 17462597

[67] Zaiss MM Axmann R Zwerina J Polzer K Gückel E Skapenko A. Treg cells suppress osteoclast formation: a new link between the immune system and bone. Arthritis Rheum. (2007) 56:4104–12. 10.1002/art.23138 18050211

[68] Zaiss MM Frey B Hess A Zwerina J Luther J Nimmerjahn F. Regulatory T cells protect from local and systemic bone destruction in arthritis. J Immunol. (2010) 184:7238–46. 10.4049/jimmunol.0903841 20483756

[69] Steinman RM Banchereau J. Taking dendritic cells into medicine. Nature. (2007) 449:419–26. 10.1038/nature06175 17898760

[70] Thomas R MacDonald KP Pettit AR Cavanagh LL Padmanabha J Zehntner S. Dendritic cells and the pathogenesis of rheumatoid arthritis. J Leukoc Biol. (1999) 66:286–92. 10.1002/jlb.66.2.286 10449169

[71] Santiago-Schwarz F Anand P Liu S Carsons SE. Dendritic cells (DCs) in rheumatoid arthritis (RA): progenitor cells and soluble factors contained in RA synovial fluid yield a subset of myeloid DCs that preferentially activate Th1 inflammatory-type responses. J Immunol. (2001) 167:1758–68. 10.4049/jimmunol.167.3.1758 11466401

[72] Rivollier A Mazzorana M Tebib J Piperno M Aitsiselmi T Rabourdin-Combe C Jurdic P Servet-Delprat C. Immature dendritic cell transdifferentiation into osteoclasts: a novel pathway sustained by the rheumatoid arthritis microenvironment. Blood. (2004) 104:4029–37. 10.1182/blood-2004-01-0041 15308576

[73] Hajishengallis G Moutsopoulos NM Hajishengallis E Chavakis T. Immune and regulatory functions of neutrophils in inflammatory bone loss. Semin Immunol. (2016) 28:146–58. 10.1016/j.smim.2016.02.002 26936034 PMC4867283

[74] Mortaz E Alipoor SD Adcock IM Mumby S Koenderman L. Update on neutrophil function in severe inflammation. Front Immunol. (2018) 9:2171. 10.3389/fimmu.2018.02171 30356867 PMC6190891

[75] Moutsopoulos NM Konkel J Sarmadi M Eskan MA Wild T Dutzan N. Defective neutrophil recruitment in leukocyte adhesion deficiency type I disease causes local IL-17-driven inflammatory bone loss. Sci Transl Med. (2014) 6:229ra40. 10.1126/scitranslmed.3007696 24670684 PMC4090608

[76] Poubelle PE Chakravarti A Fernandes MJ Doiron K Marceau AA. Differential expression of RANK, RANK-L, and osteoprotegerin by synovial fluid neutrophils from patients with rheumatoid arthritis and by healthy human blood neutrophils. Arthritis Res Ther. 9:R25. 10.1186/ar2137 17341304 PMC1906801

[77] Kong YY Yoshida H Sarosi I Tan HL Timms E Capparelli C. OPGL is a key regulator of osteoclastogenesis, lymphocyte development and lymph-node organogenesis. Nature. (1999) 397:315–323. 10.1038/16852 9950424

[78] Onal M Xiong J Chen X Thosten JD Almeida JD Manolagas SC Receptor activator of nuclear factor kB ligand (RANKL) protein expression by B lymphocytes contributes to ovariectomy-induced bone loss. J Biol Chem. (2012) 287:29851–60. 10.1074/jbc.M112.377945 22782898 PMC3436192

[79] Perlot T Penninger JM. Development and function of murine B cells lacking RANK. J Immunol. (2012) 188:1201–5. 10.4049/jimmunol.1102063 22219325

[80] Miyaura C Onoe Y Inada M Maki K Ikuta K Ito M. Increased B-lymphopoiesis by interleukin 7 induces bone loss in mice with intact ovarian functions: similarity to estrogen deficiency. Proc Natl Acad Sci USA. (1997) 94:9360–65. 10.1073/pnas.94.17.9360 9256487 PMC23193

[81] Pugliese LS Gonçalves TO Popi AF Mariano M Pesquero JB Lopes JD. B-1 lymphocytes differentiate into functional osteoclast-like cells. Immunobiology. (2012) 217:336–44. 10.1016/j.imbio.2011.07.014 21855167

[82] Manabe N Kawaguchi H Chikuda H Miyaura C Inada M Nagai R. Connection between B lymphocyte and osteoclast differentiation pathways. J Immunol. (2001) 167:2625–31. 10.4049/jimmunol.167.5.2625 11509604

[83] Söderström K Stein E Colmenero P Purath U Müller-Ladner U de Matos CT Tarner IH Robinson WH Engleman EG. Natural killer cells trigger osteoclastogenesis and bone destruction in arthritis. Proc Natl Acad Sci USA. (2010) 107:13028–33. 10.1073/pnas.1000546107 20615964 PMC2919936

[84] Feng S Madsen SH Viller NN Neutzsky-Wulff AV Geisler C Karlsson L. Interleukin-15-activated natural killer cells kill autologous osteoclasts via LFA-1, DNAM-1 and TRAIL, and inhibit osteoclast-mediated bone erosion *in vitro*. Immunology. (2015) 145:367–79. 10.1111/imm.12449 25684021 PMC4479536

[85] Ahern DJ Brennan FM. The role of Natural Killer cells in the pathogenesis of rheumatoid arthritis: major contributors or essential homeostatic modulators? Immunol Lett. (2011) 136:115–21. 10.1016/j.imlet.2010.11.001 21073898

[86] Michalski MN McCauley LK. Macrophages and skeletal health. Pharmacol Ther. (2017) 174:43–54. 10.1016/j.pharmthera.2017.02.017 28185913 PMC5429177

[87] Chitteti BR Cheng YH Poteat B Rodriguez-Rodriguez S Goebel WS Carlesso N. Impact of interactions of cellular components of the bone marrow microenvironment on hematopoietic stem and progenitor cell function. Blood. (2010) 115:3239–48. 10.1182/blood-2009-09-246173 20154218 PMC2858485

[88] Mohamad SF Xu L Ghosh J Childress PJ Abeysekera I Himes ER. Osteomacs interact with megakaryocytes and osteoblasts to regulate murine hematopoietic stem cell function. Blood Adv. (2017) 1:2520–28. 10.1182/bloodadvances.2017011304 29296903 PMC5728639

[89] Champagne CM Takebe J Offenbacher S Cooper LF. Macrophage cell lines produce osteoinductive signals that include bone morphogenetic protein-2. Bone. (2002) 30:26–31. 10.1016/S8756-3282(01)00638-X 11792561

[90] Chang MK Raggatt IJ Alexander KA Kuliwaba JS Fazzalari NL Schroder K. Osteal tissue macrophages are intercalated throughout human and mouse bone lining tissues and regulate osteoblast function *in vitro* and *in vivo*. J Immunol. (2008) 181:1232–44. 10.4049/jimmunol.181.2.1232 18606677

[91] Cho SW Soki FN Koh AJ Eber MR Entezami P Park S. Osteal macrophages support physiologic skeletal remodeling and anabolic actions of parathyroid hormone in bone. Proc Natl Acad Sci USA. (2014) 111:1545–50. 10.1073/pnas.1315153111 24406853 PMC3910564

[92] Vi L Baht GS Whetstone H Mg A Wei Q Poon R. Macrophages promote osteoblastic differentiation *in-vivo*: implications in fracture repair and bone homeostasis. J Bone Miner Res. (2015) 30:1090–102. 10.1002/jbmr.2422 25487241

[93] Gowen M MacDonald BR Russel RG. Actions of recombinant human gamma-interferon and tumor necrosis factor alpha on the proliferation and osteoblastic characteristics of human trabecular bone cells *in vitro*. Arthritis Rheum. (1988) 31:1500–7. 10.1002/art.1780311206 3143369

[94] Maruhashi T Kaifu T Yabe R Seno A Chung SH Fujikado N. DCIR maintains bone homeostasis by regulating IFN-gamma production in T cells. J Immunol. (2015) 194:5681–91. 10.4049/jimmunol.1500273 25926676

[95] Duque G Huang DC Dion N Macoritto M Rivas D Li W. Interferon-gamma plays a role in bone formation *in vivo* and rescues osteoporosis in ovariectomized mice. J Bone Miner Res. (2011) 26:1472–83. 10.1002/jbmr.350 21308779

[96] Vidal C Bermeo S Li W Huang D Kremer R Duque G. Interferon gamma inhibits adipogenesis *in vitro* and prevents marrow fat infiltration in oophorectomized mice. Stem Cells. 30:10428. 10.1002/stem.1063 22331815

[97] Ji JD Park-Min KH Shen Z Fajardo RJ Goldring SR McHugh KP. Inhibition of RANK expression and osteoclastogenesis by TLRs and IFN-gamma in human osteoclast precursors. J Immunol. (2009) 183:7223–33. 10.4049/jimmunol.0900072 19890054 PMC2783334

[98] Xiong Q Zhang I Ge W Tang P. The roles of interferons in osteoclasts and osteoclastogenesis. Joint Bone Spine. (2016) 83:276–81. 10.1016/j.jbspin.2015.07.010 26832190

[99] Kohara H Kitaura H Fujimura Y Yoshimatsu M Morita Y Eguchi T. IFN-gamma directly inhibits TNF-alpha-induced osteoclastogenesis *in vitro* and *in vivo* and induces apoptosis mediated by Fas/Fas ligand interactions. Immunol Lett. (2011) 137:53–61. 10.1016/j.imlet.2011.02.017 21338623

[100] Kim JW Lee MS Lee CH Kim HY Chae SU Kwak HB. Effect of interferon-gamma on the fusion of mononuclear osteoclasts into bone-resorbing osteoclasts. BMB Rep. (2012) 45:281–6. 10.5483/BMBRep.2012.45.5.281 22617451

[101] Kwak HB Ha H Kim HN Lee JH Kim HS Lee S. Reciprocal cross-talk between RANKL and interferon-gamma-inducible protein 10 is responsible for bone-erosive experimental arthritis. Arthritis Rheum. (2008) 58:1332–42. 10.1002/art.23372 18438854

[102] Yoshihara A Seida Y Hanada N Miyazaki H. A longitudinal study of the relationship between periodontal disease and bone mineral density in community-dwelling older adults. J Clin Periodontol. (2004) 31:680–4. 10.1111/j.1600-051X.2004.00548.x 15257747

[103] Garcia-Carrasco M Mendoza-Pinto C Escarcega RO Jimenez-Hernandez M Etchegaray Morales I Munguia Realpozo P. Osteoporosis in patients with systemic lupus erythematosus. Isr Med Assoc J. (2009) 11:486–91. 19891237

[104] Paganelli M Albanse C Borrelli O Civitelli F Canitano N Viola F. Inflammation is the main determinant of low bone mineral density in pediatric inflammatory bowel disease. Inflamm Bowel Dis. (2007) 13:416–23. 10.1002/ibd.20039 17206686

[105] Ali T Lam D Bronze MS Humphrey MB. Osteoporosis in inflammatory bowel disease. Am J Med. (2009) 122:599–604. 10.1016/j.amjmed.2009.01.022 19559158 PMC2894700

[106] Shead EF Haworth CS Barker H Bilton D Compston JE. Osteoclast function, bone turnover and inflammatory cytokines during infective exacerbations of cystic fibrosis. J Cyst Fibros. (2010) 9:93–8. 10.1016/j.jcf.2009.11.007 20006563

[107] Buckley L Humphrey MB. Glucocorticoid-induced osteoporosis. N Engl J Med. (2018) 379:2547–56. 10.1056/NEJMcp1800214 30586507

[108] Azuma Y Kaji K Katogi R Takeshita S Kudo A. Tumor necrosis factor-alpha induces differentiation of and bone resorption by osteoclasts. J Biol Chem. (2000) 275:4858–64. 10.1074/jbc.275.7.4858 10671521

[109] Komine M Kukita A Kukita T Ogata Y Hotokebuchi T Kohashi O. Tumor necrosis factor-alpha cooperates with receptor activator of nuclear factor kappaB ligand in generation of osteoclasts in stromal cell-depleted rat bone marrow cell culture. Bone. (2001) 28:474–83. 10.1016/S8756-3282(01)00420-3 11344046

[110] Matsumoto T Kuriwaka-Kido R Kondo I Kido S. Regulation of osteoblast differentiation by interleukin-11 via AP-1 and Smad signaling. Endocr J. (2012) 59:91–101. 10.1507/endocrj.EJ11-0219 21931225

[111] Suga K Saitoh M Fukushima S Takahashi K Nara H Yasuda S. Interleukin-11 induces osteoblast differentiation and acts synergistically with bone morphogenetic protein-2 in C3H10T1/2 cells. J Interferon Cytokine Res. (2001) 21:695–707. 10.1089/107999001753124435 11576464

[112] Scott DL Wolfe F Hizinga TW. Rheumatoid arthritis. Lancet. (2010) 376:1094–108. 10.1016/S0140-6736(10)60826-4 20870100

[113] Hwang SY Kim JY Kim KW Park MK Moon Y Kim WU. IL-17 induces production of IL-6 and IL-8 in rheumatoid arthritis synovial fibroblasts via NF-kappaB- and PI3-kinase/Akt-dependent pathways. Arthritis Res Ther. (2004) 6:R120–8. 10.1186/ar1038 15059275 PMC400429

[114] Ivanovic DV Di Battista JA Martel-Pelletier J Jolicoeur PC He Y Zhang M IL-17 stimulates the production and expression of proinflammatory cytokines, IL-beta and TNF-alpha, by human macrophages. J Immunol. (1998) 160:3513–21. 9531313

[115] Pacifici R Brown C Puscheck E Friedrich E Slatopolsky E Maggio D. Effect of surgical menopause and estrogen replacement on cytokine release from human blood mononuclear cells. Proc Natl Acad Sci USA. (1991) 88:5134–8. 10.1073/pnas.88.12.5134 2052592 PMC51826

[116] Hughes DE Dai A Tiffee JC Li HH Mundy GR Boyce BF. Estrogen promotes apoptosis of murine osteoclasts mediated by TGF-beta. Nat Med. (1996) 2:1132–6. 10.1038/nm1096-1132 8837613

[117] Piva R Penolazzi L Lambertini E Giordano S Gambari R. Induction of apoptosis of human primary osteoclasts treated with a transcription factor decoy mimicking a promoter region of estrogen receptor alpha. Apoptosis. (2005) 10:1079–94. 10.1007/s10495-005-0618-8 16151641

[118] Srivastava S Toraldo G Weitzmann MN Cenci S Ross FP Pacifici R. Estrogen decreases osteoclast formation by down-regulating receptor activator of NF-kappa B ligand (RANKL) induced JNK activation. J Biol Chem. (2001) 276:8836–40. 10.1074/jbc.M010764200 11121427

[119] Manolagas SC O'Brien CA Almeida M. The role of estrogen and androgen receptors in bone health and disease. Nat Rev Endocrinol. (2013) 9:699–712. 10.1038/nrendo.2013.179 24042328 PMC3971652

[120] Eghbali-Fatourechi G Khosla S Sanyal A Boyle WJ Lacey DL Riggs BL. Role of RANK ligand in mediating increased bone resorption in early postmenopausal women. J Clin Invest. (2003) 111:1221–30. 10.1172/JCI200317215 12697741 PMC152939

[121] Breuil Y Ticchioni M Testa J Roux CH Ferrari P Breittmayer JP. Immune changes in post-menopausal osteoporosis: the immunos study. Osteoporos Int. (2010) 21:805–14. 10.1007/s00198-009-1018-7 19876583

[122] D'Amelio P Grimaldi A Di Bella S Brianza SZM Cristofaro MA Tamone C. Estrogen deficiency increases osteoclastogenesis up-regulating T cells activity: a key mechanism in osteoporosis. Bone. (2008) 43:92–100. 10.1016/j.bone.2008.02.017 18407820

[123] Adeel S Singh K Vydareny KH Kumari M Shah E Weitzmann MN. Bone loss in surgically ovariectomized premenopausal women is associated with T Lymphocyte activation and thymic hypertrophy. J Investig Med. (2013) 61:1178–83. 10.2310/JIM.0000000000000016 24141238 PMC3918442

[124] Masuzawa T Miyamura C Onoe Y Kusano K Ohta H Nozawa S. Estrogen deficiency stimulates B lymphopoiesis in mouse bone marrow. J Clin Invest. (1994) 94:1090–97. 10.1172/JCI117424 8083350 PMC295170

[125] Zheng SX Vrindts Y Lopez M De Groote D Zangerle PF Collette J. Increase in cytokine production (IL-1 beta, IL-6, TNF-alpha but not IFN-gamma, GM-CSF or LIF) by stimulated whole blood cells in postmenopausal osteoporosis. Maturitas. (1997) 26:63–71. 10.1016/S0378-5122(96)01080-8 9032749

[126] Hustmyer FG Walker E Yu XP Girasole G Sakagami Y Peacock M. Cytokine production and surface antigen expression by peripheral blood mononuclear cells in postmenopausal osteoporosis. J Bone Miner Res. (1993) 8:51–9. 10.1002/jbmr.5650080108 8427049

[127] Brunetti G Colucci S Pignataro P Coricciati M Mori G Cirulli N. T cells support osteoclastogenesis in an *in vitro* model derived from human periodontitis patients. J Periodontol. (2005) 76:1675–80. 10.1902/jop.2005.76.10.1675 16253089

[128] Kawai T Matsuyama T Hosokawa Y. B and T lymphocytes are the primary sources of RANKL in the bone resorptive lesion of periodontal disease. Am J Pathol. (2006) 169:987–98. 10.2353/ajpath.2006.060180 16936272 PMC1698808

[129] Rifas L Weitzmann MN. A novel T cell cytokine, secreted osteoclastogenic factor of activated T cells, induces osteoclast formation in a RANKL-independent manner. Arthritis Rheumatol. (2009) 60:3324–35. 10.1002/art.24877 19877052 PMC2783420

[130] Weitzmann MN. T-cells and B-cells in osteoporosis. Curr Opin Endocrinol Diabetes Obes. (2014) 21:461–7. 10.1097/MED.0000000000000103 25191854 PMC4313123

[131] Richardson J Hill AM Johnston CJ McGregor A Norrish AR Eastwood D. Fracture healing in HIV-positive populations. J Bone Joint Surg Br. (2008) 90:988–94. 10.1302/0301-620X.90B8.20861 18669951

[132] El-Jawhari JJ Jones E Giannoudis PV The role of immune cells in bone haling; what we know, do not know and future perspectives. Injury. (2016) 47:2399–406. 10.1016/j.injury.2016.10.008 27809990

[133] Askalonov AA. Changes in some indices of cellular immunity in patients with uncomplicated and complicated healing of bone fractures. J Hyg Epidemiol Microbiol Immunol. (1981) 25:307–10. 7299112

[134] Timlin M Toomey D Condron C Power C Street J Murray P. Fracture hematoma is a potent proinflammatory mediator of neutrophil function. J Trauma. (2005) 58:1223–9. 10.1097/01.TA.0000169866.88781.F1 15995474

[135] Almeida CR Caires HR Vasconcelos DP Barbosa MA NAP-2 secreted by human NL cells can stimulate mesenchymal stem/stromal cell recruitment. Stem Cell Rep. (2016) 6:466–73. 10.1016/j.stemcr.2016.02.012 PMC4834048 27052313

[136] Ito H. Chemokines in mesenchymal stem cell therapy for bone repair: a novel concept of recruiting mesenchymal stem cells and possible cell sources. Mod Rheumatol. (2011) 21:113–21. 10.3109/s10165-010-0357-8 20830500

[137] Nakase T Yoshikawa H. Potential roles of bone morphogenetic proteins (BMPs) in skeletal repair and regeneration. J Bone Miner Metab. (2006) 24:425–33. 10.1007/s00774-006-0718-8 17072733

[138] Omar OM Granéli C Ekström K Karlsson C Johansson A Lausmaa J. The stimulation of an osteogenic response by classical monocyte activation. Biomaterials. (2011) 32:8190–204. 10.1016/j.biomaterials.2011.07.055 21835463

[139] Luz-Crawford P Kurte M Bravo-Alegría J Contreras R Nova-Lamperti E Tejedor G Mesenchymal stem cells regenerate a CD4^+^CD25^+^Foxp3^+^ regulatory T cell population during the differentiation process of Th1 and Th17 cells. Stem Cell Res Ther. (2013) 4:65 10.1186/scrt216 23734780 PMC3706898

[140] Akiyama K Chen C Wang D Xu X Qu C Yamaza T. Mesenchymal-stem-cell-induced immunoregulation involves FAS-ligand-/FAS-mediated T cell apoptosis. Cell Stem Cell. (2012) 10:544–55. 10.1016/j.stem.2012.03.007 22542159 PMC3348385

[141] Klyushnenkova E Mosca JD McIntosh KR Human mesenchymal stem cells suppress allogeneic T cell response *in vitro*: implications for allogenic transplantation. Blood. (1998) 92:642a−642a.

[142] Corcione A Benvenuto F Ferretti E Giunti D Cappiello V Cazzanti F. Human mesenchymal stem cells modulate B-cell functions. Blood. (2006) 107:367–72. 10.1182/blood-2005-07-2657 16141348

[143] Krevvata M Silva BC Manavalan JS Galan-Diez M Kode A Matthews BG. Inhibition of leukemia cell engraftment and disease progression in mice by osteoblasts. Blood. (2014) 124:2834–46. 10.1182/blood-2013-07-517219 25139351 PMC4314530

[144] Kode A Manavalan JS Mosialou I Bhagat G Rathinam CV Luo N. Leukaemogenesis induced by an activating β-catenin mutation in osteoblasts. Nature. (2014) 506:240–4. 10.1038/nature12883 24429522 PMC4116754

[145] Raaijmakers MH Mukherjee S Guo S Zhang S Kobayashi T Schoonmaker JA. Bone progenitor dysfunction induces myelodysplasia and secondary leukaemia. Nature. (2010) 464:852–7. 10.1038/nature08851 20305640 PMC3422863

[146] Edwards JR Sun SG Locklin R Shipman CM Adamopoulos IE Athanasou NA. LIGHT (TNFSF14), a novel mediator of bone resorption, is elevated in rheumatoid arthritis. Arthritis Rheum. (2006) 54:1451–62. 10.1002/art.21821 16649193

[147] Ishida S Yamane S Nakano S Yanagimoto T Hanamoto Y Maeda-Tanimura M. The interaction of monocytes with rheumatoid synovial cells is a key step in LIGHT-mediated inflammatory bone destruction. Immunology. (2009) 128:e315–e324. 10.1111/j.1365-2567.2008.02965.x 19019090 PMC2753922

[148] Brunetti G Faienza MF Colaianni G Gigante I Oranger A Pignataro P. Impairment of bone remodeling in LIGHT/TNFSF14-deficient mice. J Bone Miner Res. (2018a) 33:704–19. 10.1002/jbmr.3345 29178458

[149] Brunetti G Rizzi R Storlino G Bortolotti S Colaianni G Sanesi L LIGHT/TNFSF14 as a new biomarker of bone disease in multiple myeloma patients experiencing therapeutic regimens. Front Immunol. 23:2459 10.3389/fimmu.2018.02459 PMC6206078 30405638

[150] Capulli M Rufo A Teti A Rucci N Global transcriptome analysis in mouse calvarial osteoblasts highlights sets of genes regulated by modeled microgravity and identifies a “mechanoresponsive osteoblast gene signature”. J Cell Biochem. (2009) 15:240–52. 10.1002/jcb.22120 19288527

[151] Rucci N Capulli M Piperni SG Cappariello A Lau P Frings-Meuthen P Heer M Teti A. Lipocalin 2: a new mechanoresponding gene regulating bone homeostasis. J Bone Miner Res. 2015 30:357–68. 10.1002/jbmr.2341 25112732

[152] Capulli M Ponzetti M Maurizi A Gemini-Piperni S Berger T Mak TW. A complex role for Lipocalin 2 in bone metabolism: global ablation in mice induces osteopenia caused by an altered energy metabolism. J Bone Miner Res. (2018) 33:1141–53. 10.1002/jbmr.3406 29444358

[153] Costa D Principi E Lazzarini E Descalzi F Cancedda R Castagnola P. LCN2 overexpression in bone enhances the hematopoietic compartment via modulation of the bone marrow microenvironment. J Cell Physiol. (2017) 232:3077–87. 10.1002/jcp.25755 28004388

[154] Mosialou I Shikhel S Liu JM Maurizi A Luo N He Z MC4R-dependent suppression of appetite by bone-derived lipocalin 2. Nature. (2017) 16;543:385–390. 10.1038/nature21697 PMC5975642 28273060

[155] Chiu YG Ritchlin CT. Denosumab: targeting the RANKL pathway to treat rheumatoid arthritis. Expert Opin Biol Ther. (2017) 17:119–28. 10.1080/14712598.2017.1263614 27871200 PMC5794005

